# Association between overweight-obese girls and age of menarche

**DOI:** 10.1186/1687-9856-2013-S1-P94

**Published:** 2013-10-03

**Authors:** Annang Giri Moelyo, Ema Nur Fitriana

**Affiliations:** 1 Child Health Department, Faculty of Medicine, Sebelas Maret University Moewardi Hospital, Surakarta, Indonesia

## 

Menarche is the first period of menstruation in woman reproduction cycles. Several epidemiology study revealed the earlier onset of age of menarche. Better nutritional status, e.g. overweight-obese, is considered as one factor contributes to this condition. We want toknow the association between overweight-obese girls and age of menarche.

A cross sectional study was performed in SDN 1 Kleco Surakarta (elementary school) students in April 2012. All girls, aged 8-12 years old were included to this study. Nutritional status was measured anthropometically for calculating and plotting the body mass index (BMI) for age based on CDC 2000 growth chart. We concluded overweight-obese girl if BMI for age >85 percentile. An interview was done to know the age of menarche. Of 179 girls, 35 (19.6%) subjects were overweight-obese; 10 (5.7%) obese and 25 (13.9%) overweight girls. Twenty six subjects (14.5%) have had period of menarche when we did this study. Means of age of menarche is 10.72 (SD 0.89) years old; the youngest age of menarche is 9 years old (2 subjects, 7.7%). Seven subjects that had period of menache (27%) are overweight-obese. Chi-square test showed no association between overweight-obese girls and episod of age of menarche (odd ratio 1.64, 95%CI 0.63-4.39). Kruskall-Wallis test revealed no association between overweight-obese girls and means of age of menarche (p=0.906,p>0.05). This results suggest that there is no association between overweight-obese girls and means of age of menarche.

